# Self-assembled peptide-dye nanostructures for in vivo tumor imaging and photodynamic toxicity

**DOI:** 10.1038/s44303-024-00008-4

**Published:** 2024-03-04

**Authors:** Raina M. Borum, Maurice Retout, Matthew N. Creyer, Yu-Ci Chang, Karlo Gregorio, Jesse V. Jokerst

**Affiliations:** 1 Department of NanoEngineering, University of California, San Diego, La Jolla, CA 92093 USA; 2 Materials Science Department, University of California, San Diego, La Jolla, CA 92093 USA; 3 Department of BioEngineering, University of California, San Diego, La Jolla, CA 92093 USA; 4 Department of Radiology, University of California, San Diego, La Jolla, CA 92093 USA

**Keywords:** Imaging, Nanobiotechnology

## Abstract

We report noncovalent assemblies of iRGD peptides and methylene blue dyes via electrostatic and hydrophobic stacking. These resulting nanomaterials could bind to cancer cells, image them with photoacoustic signal, and then treat them via photodynamic therapy. We first assessed the optical properties and physical properties of the materials. We then evaluated their utility for live cell targeting, in vivo imaging, and in vivo photodynamic toxicity. We tuned the performance of iRGD by adding aspartic acid (DD) or tryptophan doublets (WW) to the peptide to promote electrostatic or hydrophobic stacking with methylene blue, respectively. The iRGD-DD led to 150-nm branched nanoparticles, but iRGD-WW produced 200-nm nano spheres. The branched particles had an absorbance peak that was redshifted to 720 nm suitable for photoacoustic signal. The nanospheres had a peak at 680 nm similar to monomeric methylene blue. Upon continuous irradiation, the nanospheres and branched nanoparticles led to a 116.62% and 94.82% increase in reactive oxygen species in SKOV-3 cells relative to free methylene blue at isomolar concentrations suggesting photodynamic toxicity. Targeted uptake was validated via competitive inhibition. Finally, we used in vivo bioluminescent signal to monitor tumor burden and the effect of for photodynamic therapy: The nanospheres had little impact versus controls (*p* = 0.089), but the branched nanoparticles slowed SKOV-3 tumor burden by 75.9% (*p* < 0.05).

## Introduction

New cancer therapies are urgently needed, and targeted therapies have shown value in reducing off-target effects while increasing therapeutic efficacy. Peptide-based agents have shown specific value when engineered to interact with T-cell epitopes for triggered immunogenicity^1^, upregulated membrane receptors in solid tumors^2–4^, and enzyme biomarkers for targeted inhibition. Peptides also have value when used to direct a therapeutic or diagnostic agent to the site of disease, but covalent ligation between peptides and a therapeutic or diagnostic molecular agent can suffer from modest conjugation yields, thus bottlenecking scale-up^5,6^.

Self-assembling peptide nanoparticles (SAPNs) are an important alternative to covalent chemistry when linking peptides and cargo. Here, peptide sequences are engineered so that one domain has biologically activity, i.e., targeting, while another domain tunes the peptide’s physical properties via zwitterionic, charged, or hydrophobic regimes^7,8^. Examples include tissue regeneration, targeted cytotoxicity, nucleic acid delivery, activatable immunotherapy^9^, and molecular imaging^10,11^. One limitation of SAPNs is that synthesis usually yields nanoparticles with various architectures and shapes, which can affect their interactions with biology. While many reports discuss the ideal application for different resultant geometries of SAPNs^12^, few reports empirically compare the performance of different geometries in parallel.

Here, we investigate non-covalent interactions between a tumor-homing peptide motif (iRGD) and a molecular dye (methylene blue). iRGD was chosen as the model peptide because it is a well-established and robust bimodal targeting peptide against solid tumors: iRGD first internalizes into cancer cells via α_V_β_3_ and α_V_β_5_ integrin recognition^13^. Upon cleavage into CRGDK/R, the exposed C-endR motif (R/KXXR/K) allows the fragmented peptide to enable the tumor microenvironment (TME) vasculature permeability through neuropilin-1 (NRP-1) interactions^14^. This peptide offers active targeted drug delivery by mere co-loading the peptide and the drug rather than covalent tethering^15^.

**Fig. 1:**
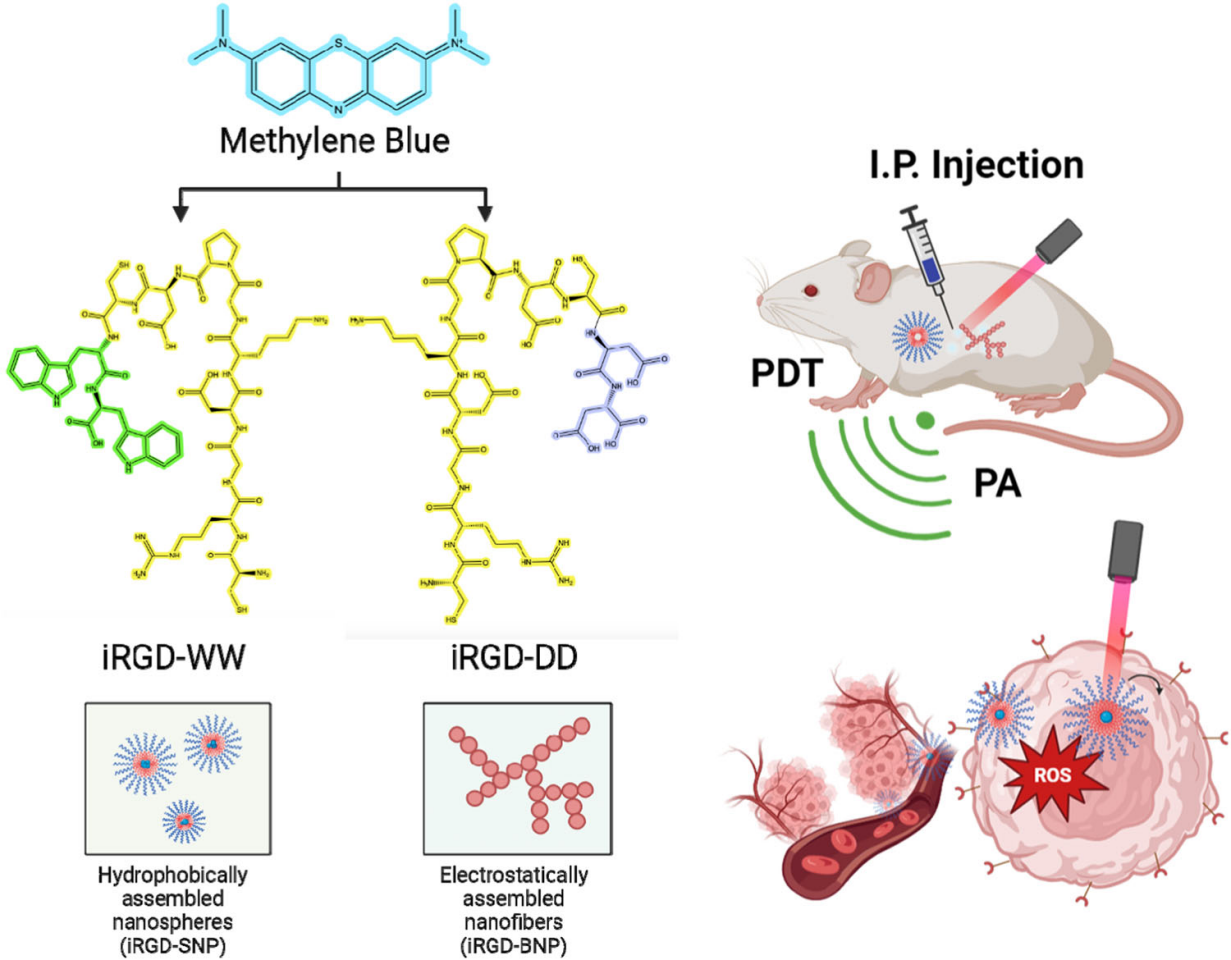
Assembly of iRGD-methylene branched NPs and nanosphere formulations based on electrostatic and hydrophobic stacking affinities. The cyan molecule is methylene blue, yellow is iRGD, green are the tryptomphan additions for iRGD-WWW and dark blue represents the two aspartic acid residues in iRGD-DD. The iRGD-methylene blue formulations were then used for in vivo tumor imaging and photodynamic toxicity.

The photodynamic therapeutic used here is methylene blue—an FDA-approved dye and longstanding workhorse in the clinical landscape; it was selected as a model molecular dye here because it is both hydrophobic and cationic, thus making it possible to explore two possible mechanisms of self-assembly. Methylene blue also absorbs light in the NIR window (~670 nm) suitable for imaging^16^. Its redox potential also enables activatable reactive oxygen species (ROS) generation for therapy (Fig. 1)^17^.

We combined the methylene blue and iRGD peptide into SAPNs to make a hybrid construct that offers targeting, therapy, and imaging. The approach used intermolecular forces rather than covalent bonds. Here, the iRGD molecule was modified with additional amino acids on the C-terminus—either aspartic acid or tryptophan. Aspartic acid (D) facilitates electrostatic interactions between iRGD and methylene blue, while tryptophan (W) produces hydrophobic stacking. These different intermolecular forces lead to different nanoparticle morphologies. After characterizing the materials’ physical properties, we then studied the value of these materials in imaging tumors with photoacoustic imaging and treating tumors with photodynamic therapy. The results show that the SAPN morphology is controllable via peptide engineering and that the product can markedly reduce tumor growth. The product offers targeting via the iRGD motif, photoacoustic imaging via the methylene blue cargo, and therapy via photodynamic therapy of the methylene blue cargo.

## Results

### Peptide design and assembly with methylene blue

Native and designer iRGD peptides were synthesized using solid phase synthesis. iRGD (CRGDKGPDC) was tagged with either aspartic acid doublets (iRGD-DD) or tryptophan doublets (iRGD-WW): We hypothesized that the negatively charged aspartic acids would facilitate electrostatic assemblies between the peptides and methylene blue; the tryptophan groups would form hydrophobic assemblies. The products were purified via reverse phase high performance liquid chromatography (RP-HPLC) and characterized via ESI-mass spectrometry (Fig. S1). Although native iRGD adopts a cyclic structure from disulfide bridging between cysteines, computational simulations demonstrated that the designer peptides do not adopt a secondary structure (Fig. S2).

**Fig. 2:**
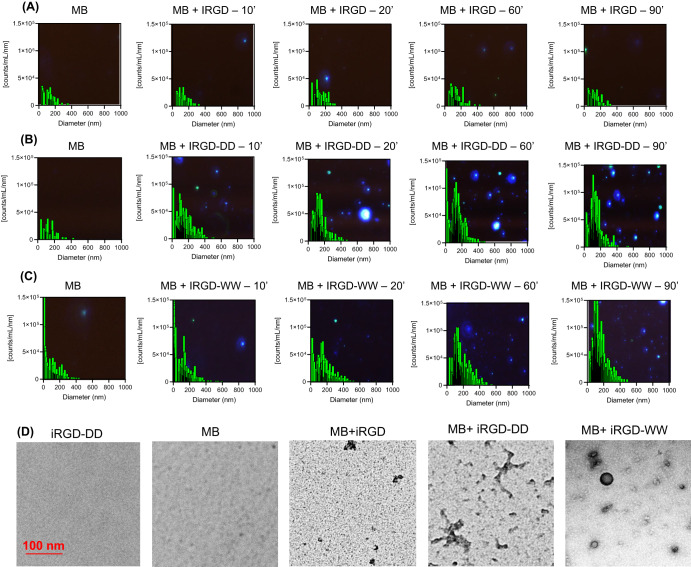
Nanoparticle characterization. **A** MANTA measurements of the native iRGD peptide with methylene blue show that no nanoparticles were formed. These data show a characteristic MANTA image (i.e., darkfield microcopy image) as the background of the plot. **B** The iRGD-DD and methylene blue form 180 nm nanoparticles over 90 minutes. **C** The iRGD-WW and methylene blue form 150 nm nanoparticles over 90 min. Data in panels **A**–**C** are overlaid with scattering light detected by the MANTA, corroborating the formation of growing nanoparticles. **D** TEM micrographs show that the iRGD-DD + methylene blue and iRGD-WW + methylene blue samples formed into branched iRGD nanoparticles (iRGD-BNPs) and spherical iRGD nanospheres (iRGD-SNPs), respectively. The molecular dye, peptides, and combination between the dye and the native iRGD did not form assemblies with characteristic shapes.

Multispectral advanced nanoparticle tracking analysis (MANTA) was used to monitor size changes and nanoparticle formations during assembly of peptide and methylene blue (Fig. 2A–C). First, native iRGD peptides were mixed with methylene blue at a 10:1 molar ratio in water. MANTA demonstrated that these mixtures did not yield any nanoparticles: The maximum average size of 9 nm, which is similar to peaks seen with native iRGD peptide in water alone (Fig. 2A).

When iRGD-DD peptides were mixed with methylene blue at the same molar ratio in water, the size increased and stabilized to about 178 nm particles within 90 min (Fig. 2B). This assembly was caused by electrostatic attraction between the aspartic acid doublets and the methylene blue. To demonstrate this, we repeated the same assembly conditions but under pH conditions that surpassed the isoelectric points of either iRGD-DD or methylene blue. When the pH was set to 12, methylene blue adopted a neutral charge, which did not result in stable nanoparticulate formations. The same results were observed when the pH was set below 2 where iRGD-DD has neutral charge. In both cases, the most abundantly observed sizes from MANTA were below 10 nm (Fig. S3A–D).

When iRGD-WW peptides were mixed with methylene blue at a similar 10:1 molar ratio in water, 150 nm nanoparticles were formed under the same 90-minute time frame (Fig. 2C). This assembly was likely due to hydrophobic stacking between the tryptophan doublets and the methylene blue molecules. To confirm this hypothesis, we studied assembly in DMSO and chloroform, which disrupt hydrophobic interactions from tryptophan. Here, no nanoparticle assembly was observed (Fig. S3E–F). Thus, both iRGD-DD and iRGD-WW based assemblies can produce SAPNs^18^.

We used transmission electron microscopy (TEM) to image the products (Fig. 2D). The individual methylene blue molecules and peptides did not show any noticeable particles. Similarly, a mixture between native iRGD and methylene blue did not reveal any visible assemblies besides some nonspecific aggregates. In contrast, the iRGD-DD-methylene blue assemblies were seen as branched nanoparticles (“iRGD-BNPs”), while iRGD-WW-methylene blue assemblies were spherical (iRGD-SNPs) (see also Fig. S4). The sizes of these particulates agreed with the MANTA measurements.

We further studied the impact of different ratios of peptides to methylene blue using MANTA analysis (Fig. S5). The number of particles produced was not significantly different than PBS background until there were two equivalents of peptide per MB for the iRGD-BNP and four equivalents of peptide per methylene blue for iRGD-SNP. Thus, all work above and below used 10 equivalents of peptide because this concentration led to the maximum particle formation (Fig. S5). Finally, we tested the stability of the products in different pH values including in physiological conditions. The results showed a slight increase at more acidic conditions typical of tumor environment (Fig. S6). The particles were also stable for up to a month when refrigerated in the dark.

#### Live cell interactions between the peptide-dye assemblies

**Fig. 3:**
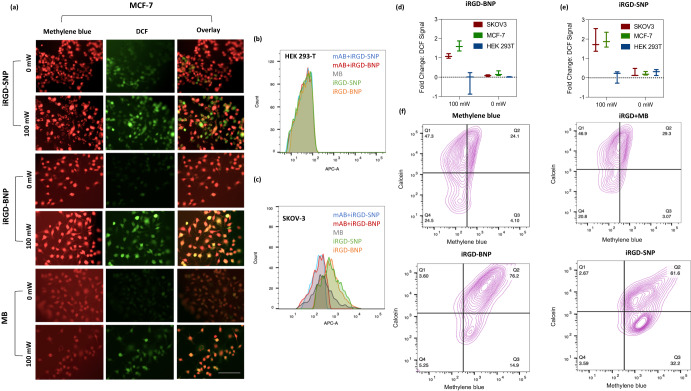
iRGD-BNP and iRGD-SNP interactions with live cells for targeted internalization and ROS generation. **a** Live cell fluorescent microscopy shows that MCF-7 cells exposed to 100 mW NIR light after incubation with the branched and the spherical nanoparticles have increased fluorescence from DCFH-DA indicating ROS generation. Cells not exposed to 100 mW NIR light do not have ROS generation (scale bar = 100 μm). **b** Competitive inhibition flow cytometry studies with HEK 293T cells shows that whether the cells are incubated with α_V_β_5_ primary antibodies before incubation, there is no change in fluorescence increase when they are treated with iRGD-BNPs, iRGD-SNPs, and methylene blue alone. **c** Competitive inhibition with SKOV-3 cells shows reduced signal upon blocking with α_V_β_5_ primary antibodies before incubation with iRGD-BNPs and iRGD-SNPs (red and blue respectively). Panels **d** and **e** show quantitative fold change in DCFH-DA-based ROS fluorescence between iRGD-BNPs and iRGD-SNPs respectively, over the DCFH-DA fluorescence from when the cells were incubated with methylene blue alone in SKOV3, MCF-7, and HEK 293T cells; note that HEK 293T does not express integrins and thus does not accumulate the nanoparticles and thus does not produce ROS. **f** Multicolor flow cytometry to monitor changes in calcein and methylene blue fluorescence show that while the cells treated with iRGD-SNPs had increased methylene blue fluorescence: The decreased population of double positive fluorescence indicates less susceptibility to endosomal escape.

We next evaluated targeted photodynamic toxicity. SKOV-3 cells were incubated with either methylene blue, a mixture of methylene blue with native iRGD, iRGD-BNPs, or iRGD-SNPs. The cells were then irradiated with a 100 mW 660 nm light to activate ROS production from the methylene blue (Fig. 3a). ROS production was monitored with a fluorescent DCFDA assay. This experiment showed the iRGD-SNPs and iRGD-BNPs produced 116.6% and 94.82% more ROS than SKOV3 cells treated with methylene blue alone, respectively (Fig. 3d, e). We also observed increased fluorescence from the DCFH-DA (153.7% and 134.2% increased fluorescence above free methylene blue for the nanospheres and branched NPs respectively) for MCF-7 human breast cancer cells (Fig. 3d, e). ROS fluorescence was lower when cells were not irradiated with light.

To further corroborate that ROS production was due to light activation and photodynamic toxicity, we next designed an area-specific light experiment: A certain area of the cell culture flask was irradiated with the NIR laser after treating with the iRGD-BNPs and iRGD-SNPs. ROS-based fluorescence was seen with fluorescent microscopy. There was more green fluorescence in the laser treated area (Fig. S7). These data confirmed that light irradiation generated ROS from MB. Hoechst staining also showed that ROS generation resulted in increased apoptosis (Fig. S7).

Both iRGD-BNPs and iRGD-SNPs formulations were then incubated with cells to evaluate targeting capabilities. We performed a control with different cells lines with differing integrin expression levels. (SKOV-3, MCF-7, and HEK 293T integrin expression levels were validated with flow cytometry experiments (Fig. S8)). The iRGD-BNPs, iRGD-SNPs, and methylene blue were incubated with HEK 293T cells as a negative control (Fig. 3d, e). There was no elevated ROS fluorescence when the HEK 293T cells were incubated with the assemblies.

The peptide formulations were further challenged via competitive inhibition flow cytometry studies to confirm their specific interactions with integrin receptors (Fig. 3b, c). In addition to labeling as before, some cells were pre-incubated with α_V_β_5_ primary antibodies before iRGD-BNPs or iRGD-SNPs. Fluorescence from the cells SKOV-3 treated with α_V_β_5_ antibodies prior to iRGD-BNPs or iRGD-SNPs was less than the fluorescence detected when they were treated with free methylene blue alone. There was increased fluorescence when the cells were treated with iRGD-BNPs or iRGD-SNPs alone. There was no observable change in fluorescence from the presence of the antibodies for HEK 293-T cells. These data confirmed that the iRGD peptide retained its α_V_β_5_ -mediated uptake into cells.

Endosomal escape has remained as a longstanding issue for nanoparticle drug delivery^19^, and past reports have also validated that the iRGD peptide inherently increases endocytosis-mediated uptake^20^. Therefore, it was important to examine if the iRGD nanospheres and branched NPs could escape the endosome. We characterized calcein leakage fluorescence by flow cytometry after co-incubating the membrane-impermeable and pH sensitive calcein dye either with iRGD-BNPs, iRGD-SNPs, methylene blue, or mixtures of iRGD and methylene blue (Fig. 3f)^21,22^. The cells incubated with the iRGD-BNPs showed 76.2% of the population was double positive in methylene blue and calcein fluorescence and 14.9% of the population was predominantly positive in methylene blue fluorescence, while the iRGD-SNPs showed 61.6% of the population was double positive in methylene blue and calcein fluorescence while 32.2% of the population was predominantly positive in methylene blue fluorescence. The results showed that while much of the methylene blue is internalized into the cells from the spherical formulation, it could not escape the endosome, unlike branched nanoparticles.

#### Optical characterization and photoacoustic imaging with the peptide dye assemblies

**Fig. 4:**
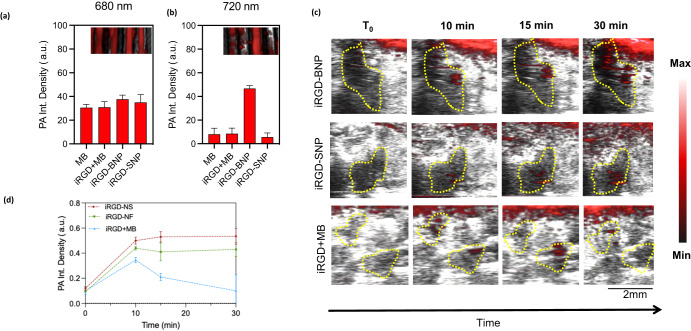
Photoacoustic characterization in vitro and in vivo. **a** Single wavelength in vitro PA scanning shows comparable PA contrast at 680 nm wavelength for all formulations tested (*p* > 0.05). **b** Single wavelength in vitro PA scanning shows sustained PA contrast from the iRGD branched NPs at 720 nm while the other formulations lost PA contrast. **c** Quantitative analysis of overlaid PA and B-mode images in (**d**) show faster rate of PA contrast in SKOV-3 i.p. murine models when injected i.p. with the iRGD-BNPs and iRGD-SNPs formulations. The yellow regions indicate regions of growing PA contrast where solid tumors are located.

The optical and photoacoustic properties of the assemblies were also studied (Fig. 4). When the nanospheres and branched NPs were scanned from 400 to 700 nm for optical absorbance, both adopted a similar peak absorbance to methylene blue at 662 nm. When the peaks were normalized, there was no 614 nm shoulder growth where dimerization typically forms^23^. While the iRGD-BNP maintained methylene blue’s distinct blue color, the iRGD-SNP samples shifted to a slightly greener color. The quantum yield for the branched NPs and the nanospheres relative to methylene blue (52%) were 43% and 31%, respectively, because there was increased fluorescence self-quenching between the assemblies relative to the methylene blue reference at the same optical density at the same wavelength (Fig. S9).

The photoacoustic contrast of the formulations was evaluated in vitro and in vivo. First, the samples were loaded into tubes, placed under water, and irradiated under a pulsed NIR laser and imaged with an ultrasound transducer. Both the nanospheres and branched NPs had photoacoustic contrast at 680 nm, which is the native peak photoacoustic intensity of methylene blue. There was no significant photoacoustic enhancement between the cases (*p* = 0.051). However, the branched NPs maintained distinct photoacoustic contrast at 720 nm while the nanospheres, mixture between methylene blue and iRGD, and methylene blue lost visibility (*p* < 0.0001) (Fig. 4a, b). Even when the iRGD-DD to methylene blue molar ratio was increased, these assemblies maintained a 720 nm photoacoustic contrast (Fig. S10). These results suggest that these non-covalent assemblies can maintain native optical properties or promote aggregation-induced shifts such as J aggregation and self-quenching based on simply tuning the peptide sequence.

Photoacoustic imaging has demonstrated ability to image diseased tissue with excellent contrast and with accessory photodynamic properties^24,25^. For the in vivo experiments, mice were xenografted with SKOV-3 cells intraperitoneally. There were six groups: (1) SKOV-3 positive and imaged with free methylene blue, (2) SKOV-3 positive and imaged with a mixture of iRGD and methylene blue, (3) SKOV-3 positive and imaged with the iRGD-BNPs, (4) SKOV-3 positive and imaged with the iRGD-SNPs, (5) SKOV-3 negative and imaged with the iRGD-BNPs, and (6) SKOV-3 negative and imaged with the iRGD-SNPs. The mice were imaged 10 days after inoculation. When the nanospheres and branched NPs were locally injected, PA contrast and intensity increased at a faster rate over the span of 15 min, while the PA contrast rate was slower when iRGD was co-injected with methylene blue.

#### Photodynamic therapy against tumor bearing mice

**Fig. 5:**
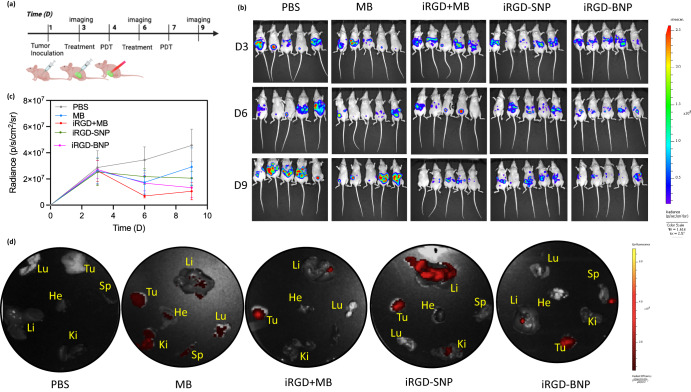
In vivo photodynamic toxicity evaluation. **a** Timeline schematic of inoculation, injection, and PDT of the subjects. Panels **b** and **c** show bioluminescent images and quantitative signal from SKOV-3 expressing luciferase in mice. Subjects treated with iRGD + MB and the iRGD branched NPs had the most decreased tumor burden. Subjects treated with iRGD nanospheres had no response. Panel **d** shows fluorescent evaluation of biodistribution, where the subjects treated with the iRGD-SNPs showed most methylene blue fluorescence in the liver despite signal from the harvested tumor as well.

Targeted photodynamic therapy against tumor-bearing mice was also evaluated. Mice were inoculated with SKOV-3 intraperitoneally, but these cells expressed the luciferase gene for bioluminescence monitoring over time. Three days after inoculation, they were treated either with (1) PBS (2) methylene blue (3) iRGD+methylene Blue, (4) iRGD-SNPs, or (5) iRGD-BNPs over the nine-day period. After overnight incubation, the subjects were exposed to a 100 mW of 660-nm light locally pointed at the abdomen for 10 min each (Fig. 5a).

Interestingly, the iRGD + methylene blue mixture and the iRGD-BNPs treatments yielded the slowest tumor growth rate, while the iRGD-NS treatment was less efficient. In particular, the branched NPs decreased tumor burden bioluminescence by an average 75.91% (+/−11.48%) (*p* = 0.043), while spheres decreased tumor burden bioluminescence by an average of 58.89% (+/−18.23%) (*p* = 0.089). There was no significant difference in the decreased bioluminescence between the branched NPs and the co-loaded iRGD and methylene blue treatment (*p* = 0.7941) (Fig. 5a–c).

The vital organs of the subjects were harvested after euthanasia and analyzed for fluorescence from the methylene blue for biodistribution studies. Here, the subjects treated with only methylene blue showed nonspecific and low fluorescence in all of the vital organs. The iRGD-SNPs-, iRGD-BNPs-, and iRGD+MB-treated subjects showed at least 2-fold heightened fluorescence in the extracted tumor. The iRGD-SNPs showed over 5-fold increased fluorescence in the liver above the other two cases (Fig. 5d). The increased fluorescence in the liver indicates preferential liver clearance of spherical nanoparticles above the branched NPs nanoparticles.

Finally, the targeting capabilities for the peptides were analyzed and compared in silico. In summary, iRGD-WW had a stronger predicted binding energy of an average docking score (affinity) of −9.82, compared to that (−8.22) of iRGD-DD to the α_V_β_3_ receptor (Figs. S11–S14). In addition, the docking ligand interactions between the peptides and receptor were compared to the ligand interactions of the co-crystallized iRGD- α_V_β_3_ complex structure (PDB 1L5G). The predicted conformations of iRGD-WW were within similar residues to iRGD’s location on the protein surface, with direct ligand interactions with directly neighboring residues to the putative binding residues of the iRGD motif (Figs. S11, S12, S14). The iRGD-DD peptide had a higher predicted affinity towards the blades of the propeller domain of the protein, which has been implicated for functionality towards the ligand binding domain (Figs. S13, S14)^26,27^. The results indicate that these peptides are capable of retaining their native affinity to the receptor.

## Discussion

Engineered peptides for SAPNs has become an increasingly popular modality to investigate due to its simplicity and multifunctionality. This work was designed to examine the extent that biomolecular targeting and supramolecular morphology contribute to enhanced uptake, contrast, and toxicity of photodynamic cargo against target-positive cells and tissue from peptide nanoparticles.

We found that the SAPNs produced between our iRGD branched and iRGD spherical nanoparticles were consistent with the literature: Electrostatically mediated peptide-small molecule assemblies preferentially adopt “checkerboard-like” patterning between complimentary ionic forces that assemble into nanofiber networks^28,29^. Hydrophobic affinities between peptides and small molecules are a well-established strategy to engineer designer vesicles, micelles, and nanoparticles^30^.

Although the native iRGD peptide typically adopts a cyclic structure due to disulfide bridging from the cysteines at either end, the engineered peptides were not cyclized during peptide synthesis to prevent adopting this conformation. The advantage here is that no higher secondary structures would hinder their self-assembly with methylene blue molecules, but it was necessary to investigate any changes in their targeting capacity. In silico docking experiments revealed that these linear peptides prefer relatively similar affinity interactions to the α_V_β_3_ protein when compared to their cyclic relative.

The modulated ROS generation and competitive inhibition studies agree with past reports that SAPNs can retain their targeting modality after assembling into higher order formulation, but the geometries of these formulations can still lead to adverse effects with live cells. Multicolor flow cytometry results show that while much of the methylene blue is internalized into the cells from the nanospherical formulation, more of the calcein dye remains trapped in the endosome with the fluorescence quenched, indicating that the fluorescence of the methylene blue in the cell can be from the dye internalized in the endosome. A couple reasons may explain the nanosphere’s entrapment: (1) nanospherical shapes have a high propensity to stay within the endosome, and (2), while hydrophobic R groups in amino acids normally aid in endosomal escape for viruses^31^. The hydrophobic stacking between the tryptophan residues and the methylene blue molecules in the nanospheres most likely suppresses their exposure for any escape capabilities for the particles.

The translation between live cell to live tissue efficacy, especially with targeted delivery, can also be compromised in a variety of ways. Although previous studies have shown that co-loaded iRGD and small molecule drug formulations have led to robust therapeutic results with simple administration, we did not expect the nanospheres to have a dampened tumor photodynamic toxicity in vivo. Nonetheless, nanoparticle formulations have shown significant impact on the pharmacokinetics and biodistribution^32^. The increased fluorescence in the liver indicates preferential liver clearance of spherical nanoparticles above the fiber nanoparticles. Liver clearance has remained as an important barrier for different kinds of nanospherical formulations, such as oncolytic viruses, lipid nanoparticles, and inorganic nanoparticles^33,34^. Strategies to overcome these barriers may include surface charge tunability and switchable self-assembly for better circulation and activated targeted delivery^35^.

We report two tailored iRGD peptides possessing either negative residues (iRGD-DD) or hydrophobic residues (iRGD-WW). These additional amino acids in turn produce electrostatic or hydrophobic interactions with methylene blue. Both peptides led to the self-assembly with the methylene blue producing nanoparticles. A mixture of MB and native iRGD did not. Assemblies of MB and our two peptides concurred with similar assemblies previously reported in the literature. Electrostatically mediated assembly results in branched nanoparticles (iRGD-BNPs). The hydrophobic affinities between peptides and MB molecules resulted in spherical nanoparticles (iRGD-SNPs). Native iRGD peptides did not produce assemblies. The product offers targeting via the iRGD sequence, photoacoustic contrast via the methylene blue cargo, and cytotoxicity via photodynamic therapy of the methylene blue cargo. Future work will integrate additional peptide structures to further refine the morphology of the particles for improved in vivo targeting capabilities.

## Methods

### Peptide synthesis and preparation

Peptides were synthesized using standard solid-phase synthesis (AAPTEC Eclipse) on Wang resin solid support. Peptides were cleaved with TFA:Phenol:Water:Thioanisole:EDOT (82.5:2.5:5:5:5) and washed thrice with cold diethyl ether. After lyophilization, peptides were resuspended in water and acetonitrile (15%), and then purified via reverse phase high performance liquid chromatography (RP-HPLC) with a Shimadzu LC-40 HPLC system equipped with a LC-40D solvent delivery module, photodiode array detector SPD-M40, and degassing unit DGU-403, using a gradient of approximately 1% of acetonitrile per minute. Pure fractions were characterized via ESI-Mass Spectrometry, and further lyophilized before use.

### Peptide dye assemblies

Peptides were mixed with methylene blue in pure milliQ water at a 10:1 peptide-dye molar ratio unless specified. They were allowed to react at 250 rpm in the dark for two hours.

### Multispectral Nanoparticle Tracking Analysis

MANTA ® Multispectral Nanoparticle Tracking Analysis (Horiba Scientific, Irvine, CA) was used for size and concentration measurements of nanoparticles and particle formation kinetics. Before each sample was measured, a blank measurement of the reaction solvent was measured to reduce background.

### Cell culture

Human ovarian adenocarinoma (SKOV-3), epithelial human breast cancer (MCF-7), and human embryonic kidney cells (HEK293-T) were used for the cell experiments. SKOV-3 was cultured in McCoy’s 5 A Medium, and MCF-7 and HEK 293T cells were cultured in DMEM. Medium was supplemented with 10% fetal bovine serum (FBS) and 1% penicillin/streptomycin. Cells passaged three times before use.

### In vitro photodynamic cytotoxicity

Cells were seeded overnight in 96 well plates at 10,000 cells per well. The 100 nM methylene blue and peptide-methylene blue assemblies were then incubated with the cells for two hours before rinsing with PBS. The amount of methylene blue was held constant for all cases for comparison. For irradiation, a NIR laser (660 nm, 100 mW cm^−2^) was fixed on a ring stand and pointed to the well for 10 minutes before characterization. Dark control samples were seeded on an entirely different plate and were kept in the dark. Cytotoxicity analysis used a resazurin assay where 10 μL of dye was added to each well after irradiation. The cells were then incubated overnight with the resazurin before measuring color changes.

### In vitro ROS cytotoxicity

For ROS assays, 10 µM DCFH-DA dye was incubated with the cells for one hour before adding 100 nM peptide-dye or dye-only with two hours of incubation. The cells were rinsed in fresh PBS twice and then irradiated to induce photothermal therapy. Hoechst 33342 NucBlue (ThermoFisher) was added to the cells for nuclei staining.

### Integrin validation

300,000 cells were detached with trypsin, and then washed in 1 mL ice cold buffer (PBS with 5% BSA and 0.1% sodium azide) at 700 × *g*. The cells were then incubated with the primary α_V_β_3_ (LM609) and α_V_β_5_ (P1F6) mAB antibodies (20 µg/µL) (abcam) on ice for 30 min. After being washed twice, the cells were incubated with goat anti-mouse IgG Alexa Fluor 488 for 30 min on ice in the dark. The cells were then washed twice and resuspended in 1 mL of ice-cold buffer before flow cytometry was performed using a FITC channel.

### Competitive inhibition

300,000 cells were seeded overnight. For the competitive inhibition samples, cell cultures were first treated with α_V_β_5_ mABs (20 µg/µL) for 90 min. The cells were then treated with the iRGD-BNPs, iR GD-SNPs, or methylene blue. The cells were detached, collected at 700xG, and the media was decanted while the cells were resuspended in ice cold buffer for flow cytometry using the APC channel.

### Endosomal escape

300,000 cells per culture were seeded overnight. The following day, the cells were incubated with the iRGD-BNPs, iRGD-NSs, methylene blue, and the iRGD methylene blue mixture for 90 min before incubation with calcein for one hour. The cells were then rinsed, detached with trypsin, then resuspended in ice cold buffer for flow cytometry. The FITC and APC channels were used.

### Photoacoustic imaging

PA images of in vitro samples were acquired with a Vevo system (VisualSonics) using a 21 MHz transducer (LZ-250). Samples were loaded into 0.86 mm polyethylene tubes and fixed in parallel with a 3D printed sample holder. One tube was filled with reaction solvent to serve as a reference. The fixed samples were placed 1 cm below the transducer in a vessel filled with water. Single wavelength scans were operated at 680 and 720 nm at a frame rate of 20 Hz. For 3D PA images, the transducer was scanned with a stepper motor along the axial dimension of the tubes. PA spectra were taken from 680 to 900 nm with a step size of 2 nm.

### Animal studies

All mice studies described below were performed in accordance with National Institutes of Health (NIH) Guidelines approved by the Institutional Animal Care and Use Committee (IACUC) under protocol S15050 at University of California, San Diego. Female J:NU mice of 5 weeks of age were used for all in vivo experiments. For imaging experiments, mice were anesthetized with 1–2% isoflurane.

### In vivo photoacoustic imaging

Mice were divided into six groups of three and inoculated with 800,000 SKOV3 (with 50% Matrigel/PBS v/v) intraperitoneally on the right side at the second nipple from the lower limbs. Ten days after inoculation, the mice were intraperitoneally injected with MB, iRGD+MB, iRGD-BNPs, iRGD-Ns at 9 mg/kg. The negative controls were not inoculated with SKOV3 cells but were injected with the iRGD-NS at the same concentration. During imaging, the animals were anesthetized with isoflurane (1–2%) and laid supine on a heated imaging stage and the transducer was directly placed above the injection site. Subjects injected with MB, iRGD-NS, and iRGD+MB were imaged using 680 nm wavelength while the subject injected with iRGD-BNPs was imaged using 720 nm wavelength. All mice were imaged under the same PA and ultrasound gain.

### In vivo bioluminescence

Mice were divided into five groups of five. Here, 800,000 SKOV3-luc cells were injected (with 50% Matrigel/PBS v/v) intraperitoneally on the right side at the second nipple from the bottom. On days 3 and 6, the mice were intraperitoneally injected with MB, iRGD+MB, iRGD-SNPs, or iRGD-BNPs (all at 9 mg/kg) or PBS. To measure tumor burden, mice were imaged with D-luciferin on days 3, 6, and 9, with 100 mg/kg dosage in PBS. The bioluminescence was measured and imaged via IVIS Perkin-Elmer Illumination and LivingImage software.

### Ex vivo

After the subject was euthanized, the heart, lungs, liver, spleen, kidneys, and tumor were harvested and rinsed in PBS. The organs were then imaged for fluorescence signal via IVIS Perkin-Elmer Illumination and LivingImage software.

### In silico experiments

Molecular Operating Environment (Chemical Computing Group) was used to predict docking interactions between our peptides and α_V_β_3_. The α_V_β_3_ – RGD complex (PDB 1L5G) was first added into MOE, and quick prepped to an RMS gradient of 0.01 kcal/mol/A^2^. Peptide structures for iRGD-DD and iRGD-WW were comprised of the molecular database made to predict and compare their docking interactions. The database was washed and quick prepped before docking simulations. We used induced fit for method refinement, with five poses predicted for each peptide. Docking potentials, predicted images of the complexes, and ligand interactions were analyzed.

## Supplementary information

Supplementary Information

## Data Availability

Data used and analyzed during the study are available from the corresponding author on reasonable request.

## References

[1] Malonis, R. J., Lai, J. R. & Vergnolle, O. Peptide-based vaccines: current progress and future challenges. *Chem. Rev.* **120**, 3210–3229 (2020). 31804810 10.1021/acs.chemrev.9b00472 PMC7094793

[2] Aloisio, A. et al. Phage-displayed peptides for targeting tyrosine kinase membrane receptors in cancer therapy. *Viruses* **13**, 649 (2021). 33918836 10.3390/v13040649 PMC8070105

[3] Boohaker, R. J., Lee, M. W., Vishnubhotla, P., Perez, J. M. L. & Khaled, A. R. The use of therapeutic peptides to target and to kill cancer cells. *Curr. Med. Chem.* **19**, 3794–3804 (2012). 22725698 10.2174/092986712801661004 PMC4537071

[4] Wang, Z. et al. Airway administration of bisphosphate and dexamethasone inhibits SARS-CoV-2 variant infection by targeting alveolar macrophages. *Signal Transduct. Target Ther.* **7**, 22–25 (2022). 35387969 10.1038/s41392-022-00977-1 PMC8984664

[5] Fitzgerald, M. C. & West, G. M. Painting proteins with covalent labels: what’s in the picture? *J. Am. Soc. Mass. Spectrom.* **20**, 1193–1206 (2009). 19269190 10.1016/j.jasms.2009.02.006

[6] Zegota, M. M. et al. Dual stimuli-responsive dynamic covalent peptide tags: toward sequence-controlled release in tumor-like microenvironments. *J. Am. Chem. Soc.* **143**, 17047–17058 (2021). 34632780 10.1021/jacs.1c06559 PMC8532147

[7] Li, S. et al. Smart peptide-based supramolecular photodynamic metallo-nanodrugs designed by multicomponent coordination self-assembly. *J. Am. Chem. Soc.* **140**, 10794–10802 (2018). 30102029 10.1021/jacs.8b04912

[8] Chang, R., Zhao, L., Xing, R., Li, J. & Yan, X. Functional chromopeptide nanoarchitectonics: molecular design, self-assembly and biological applications. *Chem. Soc. Rev.* **52**, 2688–2712 (2023). 36987746 10.1039/d2cs00675h

[9] Li, S. et al. Supramolecular nanofibrils formed by coassembly of clinically approved drugs for tumor photothermal immunotherapy. *Adv. Mater.* **33**, 1–9 (2021). 10.1002/adma.202103733 34288153

[10] Boisguérin, P., Konate, K., Josse, E., Vivès, E. & Deshayes, S. Peptide-based nanoparticles for therapeutic nucleic acid delivery. *Biomedicines* **9**, 583 (2021). 34065544 10.3390/biomedicines9050583 PMC8161338

[11] Doll, T. A. P. F., Dey, R. & Burkhard, P. Design and optimization of peptide nanoparticles. *J. Nanobiotechnol.* **13**, 1–12 (2015). 10.1186/s12951-015-0119-z PMC4619341 26498651

[12] Sun, L., Zheng, C. & Webster, T. J. Self-assembled peptide nanomaterials for biomedical applications: promises and pitfalls. *Int. J. Nanomed.* **12**, 73–86 (2017). 10.2147/IJN.S117501 PMC5191618 28053525

[13] Sugahara, K. N. et al. Tumor-penetrating IRGD peptide inhibits metastasis. *Mol. Cancer Ther.* **14**, 120–128 (2015). 25392370 10.1158/1535-7163.MCT-14-0366 PMC4297196

[14] Kang, S., Lee, S. & Park, S. IRGD peptide as a tumor-penetrating enhancer for tumor-targeted drug delivery. *Polymers* **12**, 1906 (2020). 32847045 10.3390/polym12091906 PMC7563641

[15] Sugahara, K. et al. Coadministration of a tumor-penetrating peptide enhances the efficacy of cancer drugs. *Science* **328**, 1031–1036 (2010). 20378772 10.1126/science.1183057 PMC2881692

[16] Jeon, M. et al. Methylene blue microbubbles as a model dual-modality contrast agent for ultrasound and activatable photoacoustic imaging. *J. Biomed. Opt.* **19**, 016005 (2014). 10.1117/1.JBO.19.1.016005 24390438

[17] Shen, J. J., Arendrup, M. C., Jemec, G. B. E. & Saunte, D. M. L. Photodynamic therapy: a treatment option for terbinafine resistant trichophyton species. *Photodiagnosis Photodyn. Ther.* **33**, 102169 (2021). 33497815 10.1016/j.pdpdt.2020.102169

[18] Chang, R. et al. Amino-acid-encoded supramolecular photothermal nanomedicine for enhanced cancer therapy. *Adv. Mater.* **34**, 1–9 (2022). 10.1002/adma.202200139 35178775

[19] Wu, P., Yin, S., Liu, T., Ding, D. & Wang, K. “Building-block crosslinking” micelles for enhancing cellular transfection of biocompatible polycations. *Sci. China Mater.* **64**, 241–251 (2021).

[20] Chen, B. et al. IRGD tumor-penetrating peptide-modified nano-delivery system based on a marine sulfated polysaccharide for enhanced anti-tumor efficiency against breast cancer. *Int. J. Nanomed.* **17**, 617–633 (2022). 10.2147/IJN.S343902 PMC8842734 35173433

[21] Akasov, R. et al. Formation of multicellular tumor spheroids induced by cyclic RGD-peptides and use for anticancer drug testing in vitro. *Int. J. Pharm.* **506**, 148–157 (2016). 27107900 10.1016/j.ijpharm.2016.04.005

[22] Hausig-Punke, F., Richter, F., Hoernke, M., Brendel, J. C. & Traeger, A. Tracking the endosomal escape: a closer look at calcein and related reporters. *Macromol. Biosci.* **22**, 1–26 (2022). 10.1002/mabi.202200167 35933579

[23] Wang, J., Lin, C. Y., Moore, C., Jhunjhunwala, A. & Jokerst, J. V. Switchable photoacoustic intensity of methylene blue via sodium dodecyl sulfate micellization. *Langmuir* **34**, 359–365 (2018). 29232146 10.1021/acs.langmuir.7b03718 PMC6200325

[24] Lv, J., Xu, Y., Xu, L. & Nie, L. Quantitative functional evaluation of liver fibrosis in mice with dynamic contrast-enhanced photoacoustic imaging. *Radiology* **300**, 89–97 (2021). 33904773 10.1148/radiol.2021204134

[25] Chen, R. et al. Photoacoustic molecular imaging-escorted adipose photodynamic-browning synergy for fighting obesity with virus-like complexes. *Nat. Nanotechnol.* **16**, 455–465 (2021). 33526836 10.1038/s41565-020-00844-6

[26] Xiong, J. P. et al. Crystal structure of the extracellular segment of integrin alpha Vbeta 3 in complex with an Arg-Gly-Asp ligand. *Science* **296**, 151–155 (2002). 11884718 10.1126/science.1069040

[27] Pons, T., Gómez, R., Chinea, G. & Valencia, A. Beta-propellers: associated functions and their role in human diseases. *Curr. Med. Chem.* **10**, 505–524 (2003). 12570695 10.2174/0929867033368204

[28] Lee, S. et al. Self-assembling peptides and their application in the treatment of diseases. *Int. J. Mol. Sci.* **20**, 5850 (2019). 31766475 10.3390/ijms20235850 PMC6928719

[29] Zhang, S., Holmes, T., Lockshin, C. & Rich, A. Spontaneous assembly of a self-complementary oligopeptide to form a stable macroscopic membrane. *Proc. Natl Acad. Sci. USA* **90**, 3334–3338 (1993). 7682699 10.1073/pnas.90.8.3334 PMC46294

[30] Bellomo, E. G., Wyrsta, M. D., Pakstis, L., Pochan, D. J. & Deming, T. J. Stimuli-responsive polypeptide vesicles by conformation-specific assembly. *Nat. Mater.* **3**, 244–248 (2004). 15034560 10.1038/nmat1093

[31] Lönn, P. et al. Enhancing endosomal escape for intracellular delivery of macromolecular biologic therapeutics. *Sci. Rep.* **8**, 32301 (2016). 10.1038/srep32301 PMC5015074 27604151

[32] Tsoi, K. M. et al. Mechanism of hard-nanomaterial clearance by the liver. *Nat. Mater.* **15**, 1212–1221 (2016). 27525571 10.1038/nmat4718 PMC5132626

[33] Li, Y., Duan, H. Y., Yang, K. D. & Ye, J. F. Advancements and challenges in oncolytic virus therapy for gastrointestinal tumors. *Biomed. Pharmacother.* **168**, 115627 (2023). 37812894 10.1016/j.biopha.2023.115627

[34] Blanco, E., Shen, H. & Ferrari, M. Principles of nanoparticle design for overcoming biological barriers to drug delivery. *Nat. Biotechnol.* **33**, 941–951 (2015). 26348965 10.1038/nbt.3330 PMC4978509

[35] He, S. et al. Charge-reversal polymer nano-modulators for photodynamic immunotherapy of cancer. *Angew Chem. Int. Ed. Engl.* **60**, 19355–19363 (2021). 34105217 10.1002/anie.202106392

