# Supplementary material for: Self-assembled peptide-dye nanostructures for in vivo tumor imaging and photodynamic toxicity
Source: Npj Imaging. 2024 Mar 4;2:4. doi: 10.1038/s44303-024-00008-4 (PMC12118722; doi:10.1038/s44303-024-00008-4)
Supplement: Supplementary file 1 — Supplementary Information [file 44303_2024_8_MOESM1_ESM.pdf]

Supporting information to:

Self-assembled peptide-dye nanostructures for in vivo tumor imaging and photodynamic toxicity

Raina M. Borum[1], Maurice Retout[1], Matthew N. Creyer[1], Yu-Ci Chang[3], Karlo Gregorio[4], Jesse V. Jokerst*[1,2,3]

1 Department of NanoEngineering, University of California, San Diego, La Jolla, CA, USA 92093
2 Department of Radiology, University of California, San Diego, La Jolla, CA, USA 92093
3 Materials Science Department, University of California, San Diego, La Jolla, CA, USA, 92093
4 Department of BioEngineering, University of California, San Diego, La Jolla, CA, USA 92093
*Jesse V. Jokerst.
Email: jjokerst@eng.ucsd.edu

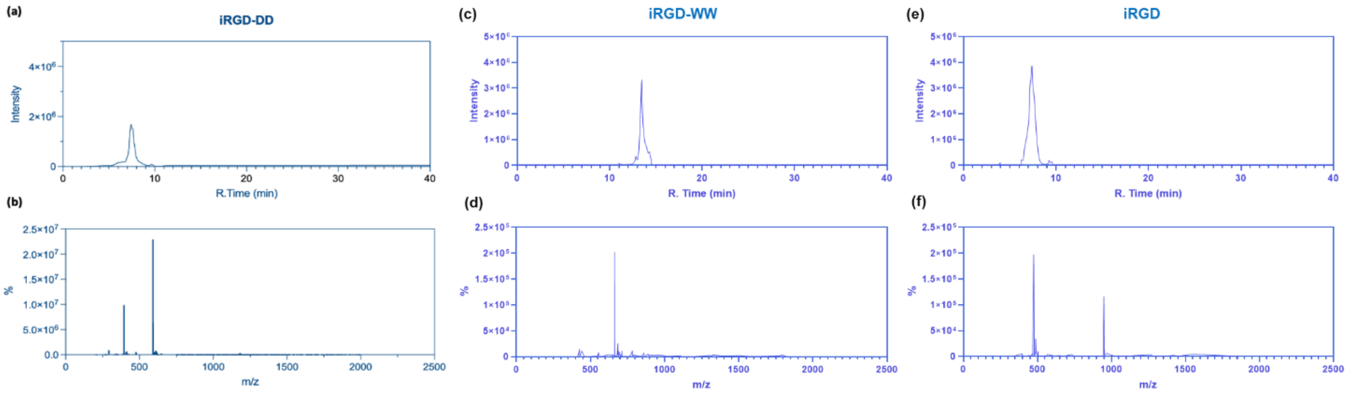

Figure S1. Chromatograms and ESI mass spectra of the peptides. (a) HPLC chromatogram of pure iRGD-DD peptide. **(b)** ESI mass spectra of the product. Expected m/z: 1180.43, 590.72, 394.15 for +1, +2, and +3 charges, respectively. Found: 1180.74, 590.25, 394.16. **(c)** HPLC chromatogram of pure iRGD-WW peptide. **(d)** ESI mass spectra of the product. Expected m/z: 660.76 +2 charges. Found: 661.21. **(e)** HPLC chromatogram of pure iRGD peptide. **(f)** ESI mass spectra of the product. Expected m/z: 949.37, 474.68 for +1 and +2 charges. Found: 949.86 and 475.59.

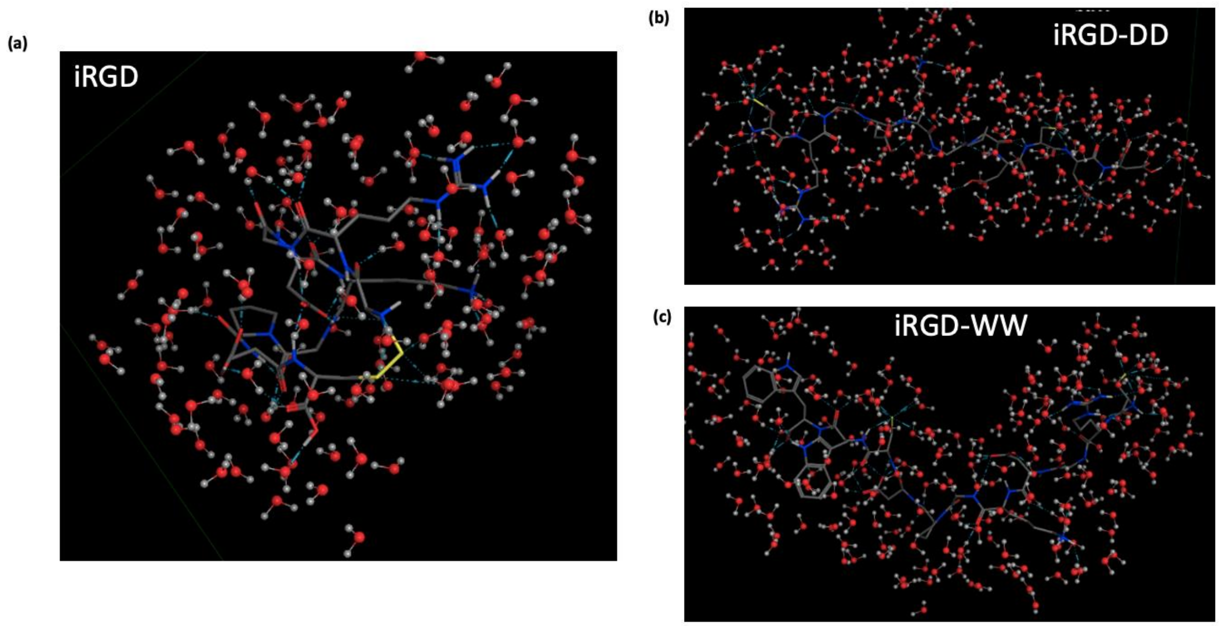

Figure S2. Simulations of the iRGD and designer iRGD peptide structures using MOE.

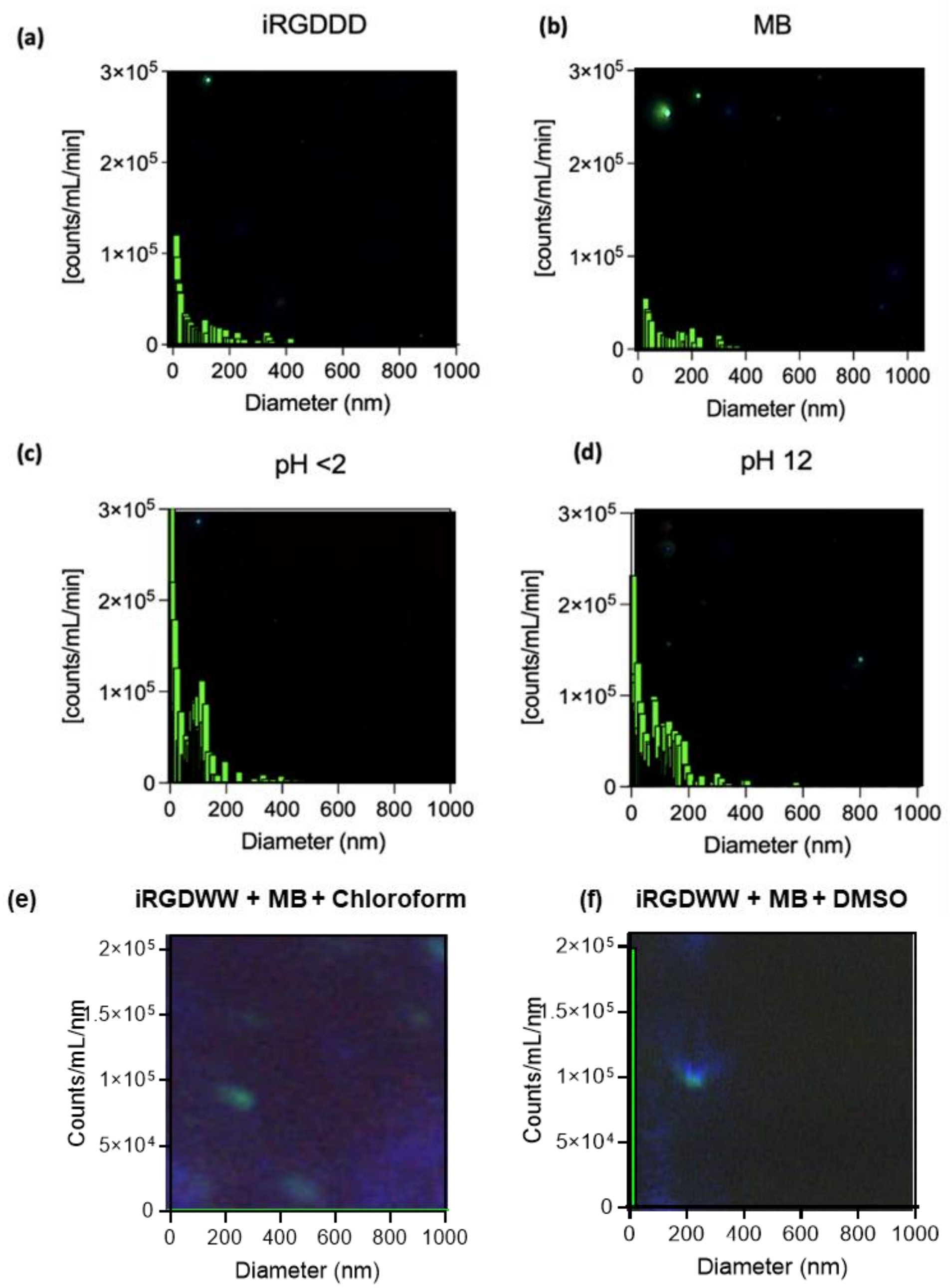

Figure S3. Control MANTA experiments. These data show a representative MANTA image (i.e., darkfield microcopy image) as the background of the plot. The peptide only **(a)** and methylene blue only **(b)** do not produce nanoparticles unlike a mixture of the two shown in Figure 1. The data in panels **(c)** and **(d)** use different pH solvents to change the charge of the iRGDDD peptide and methylene blue. This also prevents particle assembly. Panels **(e)** and **(f)** show that assembly of iRGD-WW and methylene blue does not occur in organic solvent because their driving assembly force is hydrophobic stacking. Note: The iRGD-DD-methylene blue assemblies form branched nanoparticles (iRGD-BNPs) while iRGD-WW-methylene blue assemblies were spherical (iRGD-SNPs).

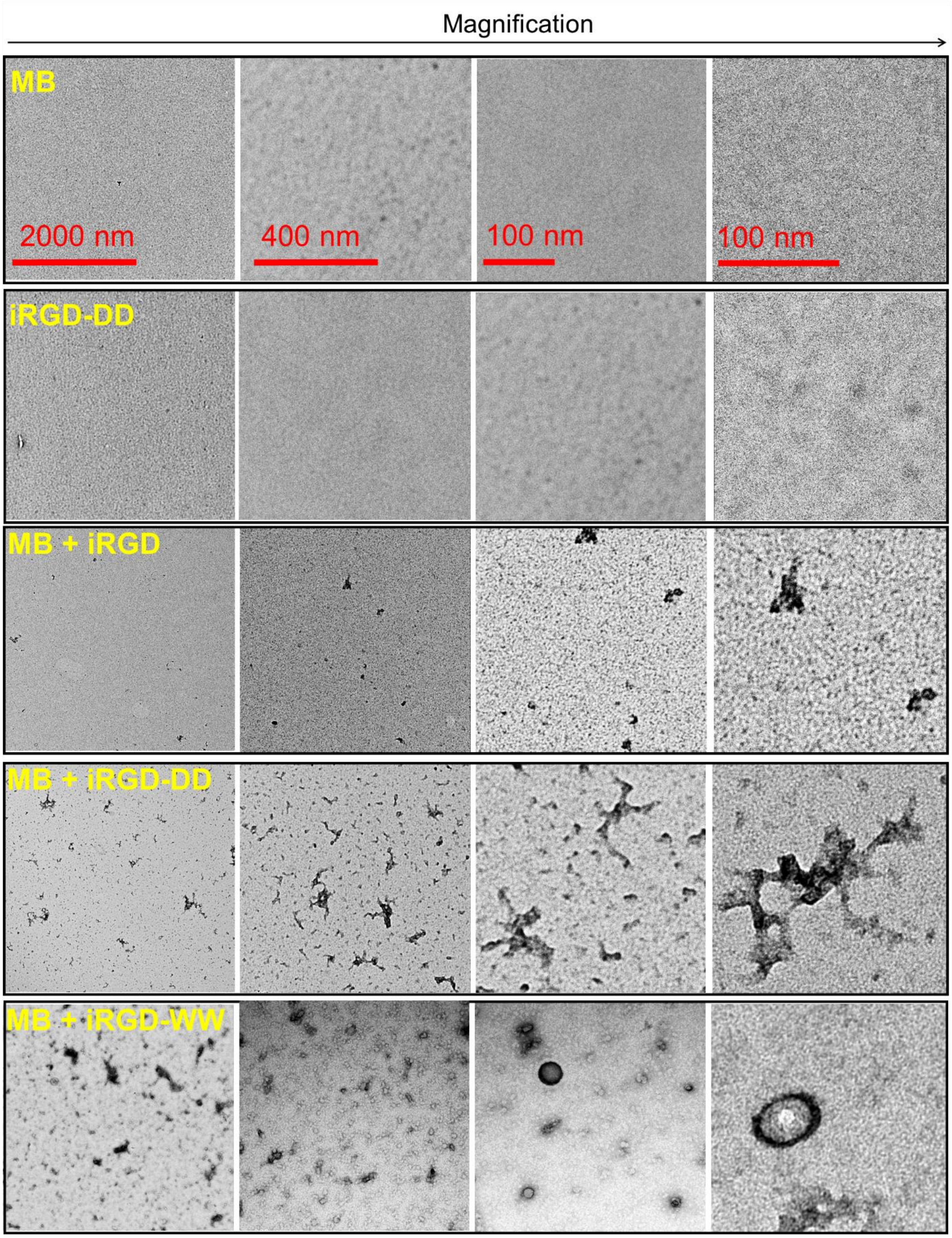

Figure S4. TEM micrographs of the assemblies with increased magnification. Note: The iRGD-DD-methylene blue assemblies form branched nanoparticles (iRGD-BNPs) while iRGD-WW-methylene blue assemblies were spherical (iRGD-SNPs).

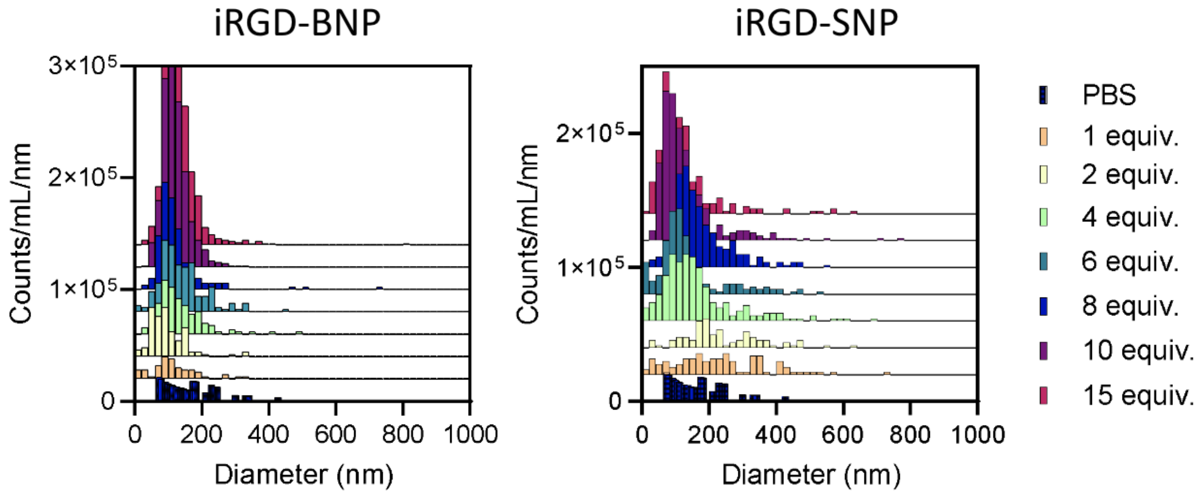

Figure S5. Titration of the peptide to MB solution. For iRGD-DD we observed the formation of branched nanoparticles (iRGD-BNP) at two equivalents of peptide per MB. For iRGD-WW, we observed the formation of spherical nanoparticles (iRGD-SNP) starting with four equivalents of peptide per MB. For this study, we chose 10 equiv. of peptide as this concentration lead to the maximum particle formation.

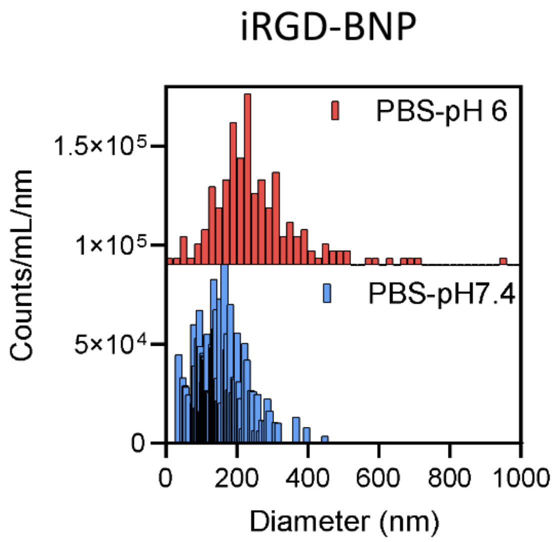

Figure S6. Formation of the iRGD-BNP at pH 7.4 or pH 6 in PBS. We observed the formation of both kind of particles in physiological condition (i.e., PBS pH7.4). The tumor can have acidic pH, and we also tested the formation of the particles that are the most sensitive to pH variation (iRGD-BNP) because they are assembled via electrostatic interactions. The iRGD-BNPs still formed even at pH = 6.

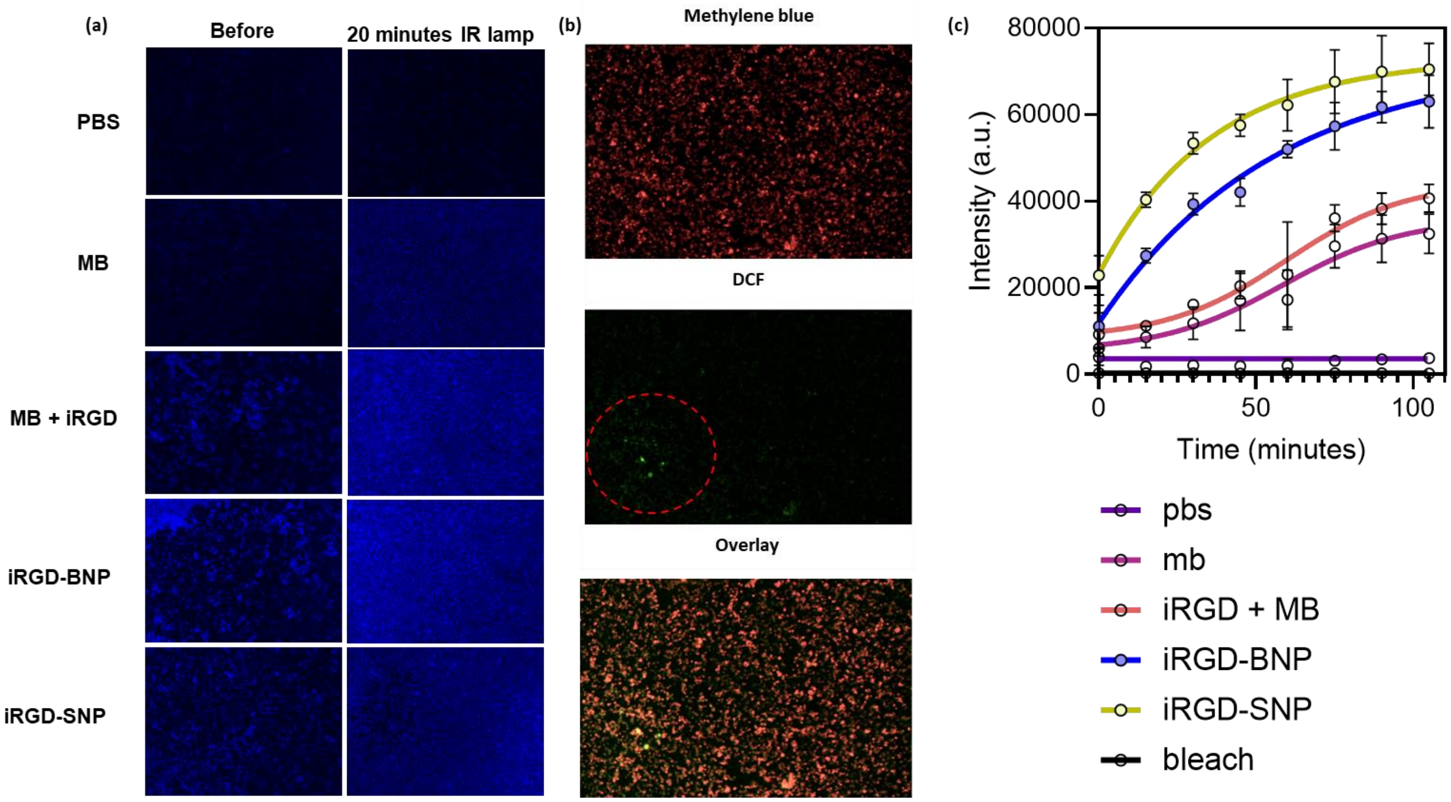

Figure S7. Validation of photodynamic therapy. (a) NucBlue stain shows increasing apoptosis activity after light irradiation when cells are incubated with the iRGD-BNPs and iRGD-SNPs assemblies. Panel **(b)** shows microscopy of DCF fluorescence elevated where flask was exposed to the red laser (denoted by the red circle in the DCF fluorescence micrograph). **(c)** Time-dependent ROS generation shows the heightened ROS fluorescence from cells incubated with the iRGD-BNPs and iRGD-SNPs (blue and yellow, respectively). ROS generation is more pronounced than iRGD+MB and MB alone (orange and red respectively). Note: The iRGD-DD-methylene blue assemblies form branched nanoparticles (iRGD-BNPs) while iRGD-WW-methylene blue assemblies were spherical (iRGD-SNPs).

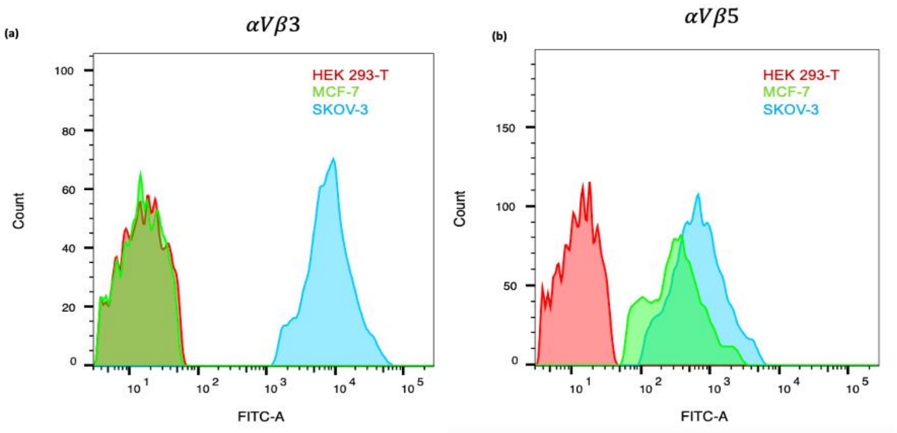

Figure S8. Integrin expression validation of the tested cell lines using Alexa Fluor 488 tagged antibodies.

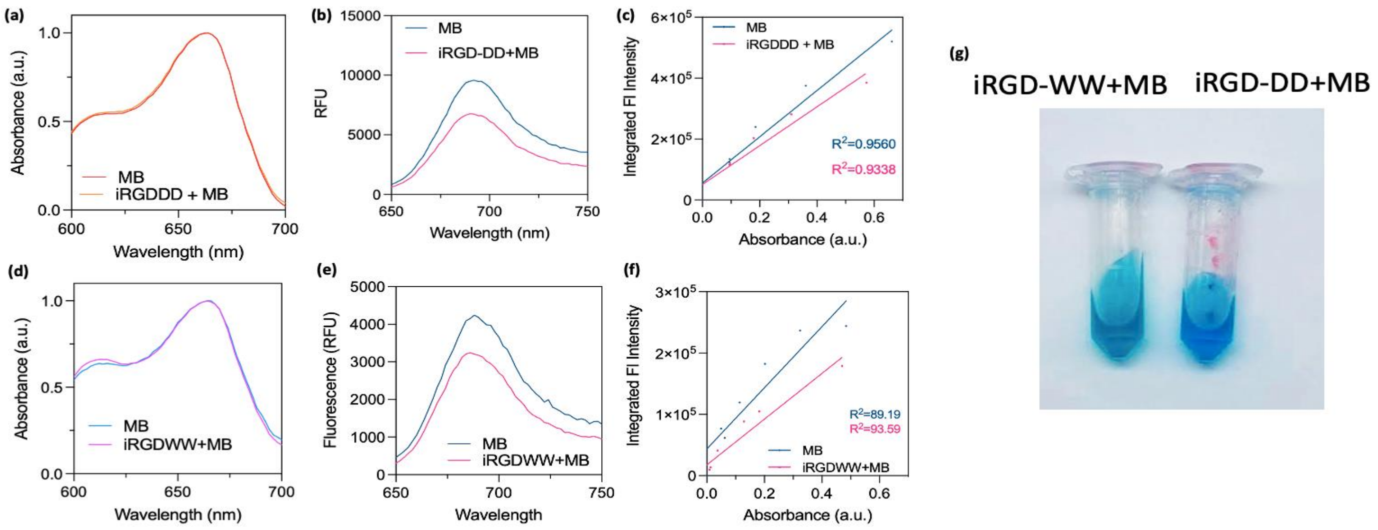

Figure S9 Optical absorbance measurements. Panels **(a-c)** show no 614 nm optical shoulder growth between the iRGD-DD-mediated assemblies, but increased fluorescent quenching at the same concentration with a lower quantum yield relative to methylene blue. Panels **(d-f)** show no 614 nm optical shoulder growth between the iRGD-WW mediated assemblies, but increased fluorescent quenching at the same concentration with a lower quantum yield relative to methylene blue. **(g)** Photograph of the assemblies. There is a green tint for the iRGD-WW mediated formation. The iRGD-DD-methylene blue assemblies form branched nanoparticles (iRGD-BNPs) while iRGD-WW-methylene blue assemblies were spherical (iRGD-SNPs).

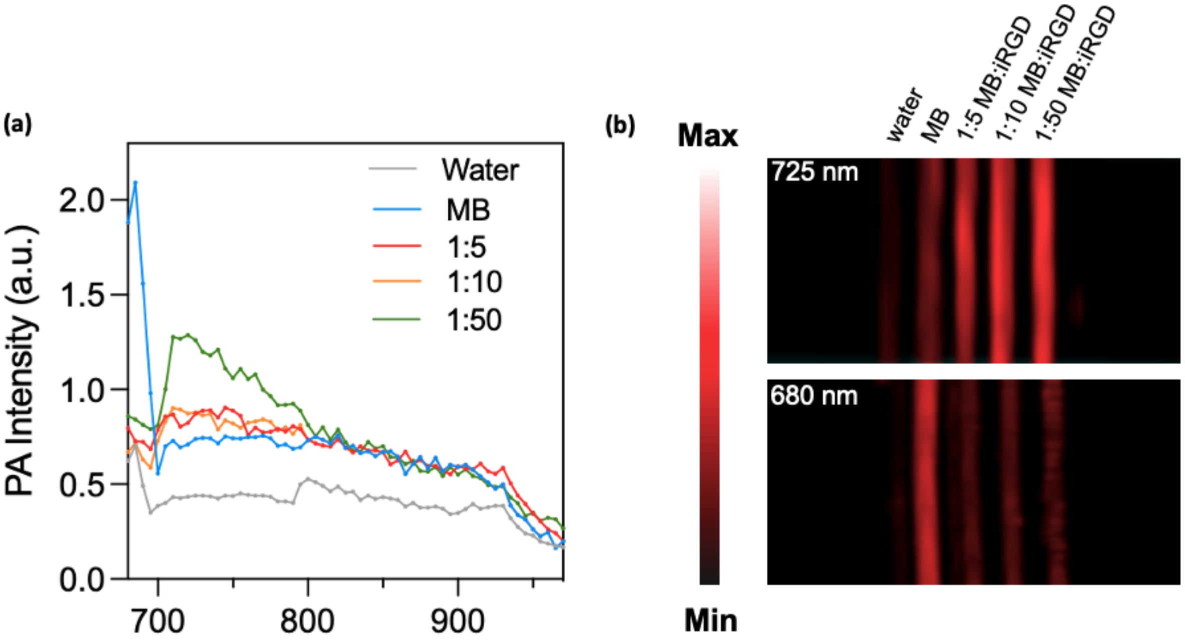

Figure S10. Photoacoustic data. The iRGD-BNPs has elevated photoacoustic signal at 725 nm excitation. Panel **(a)** shows a photoacoustic spectra for the maximum intensity projections in **(b)**. The iRGD-BNP maintain consistent photoacoustic signal beyond methylene blue's signature 680 nm peak.

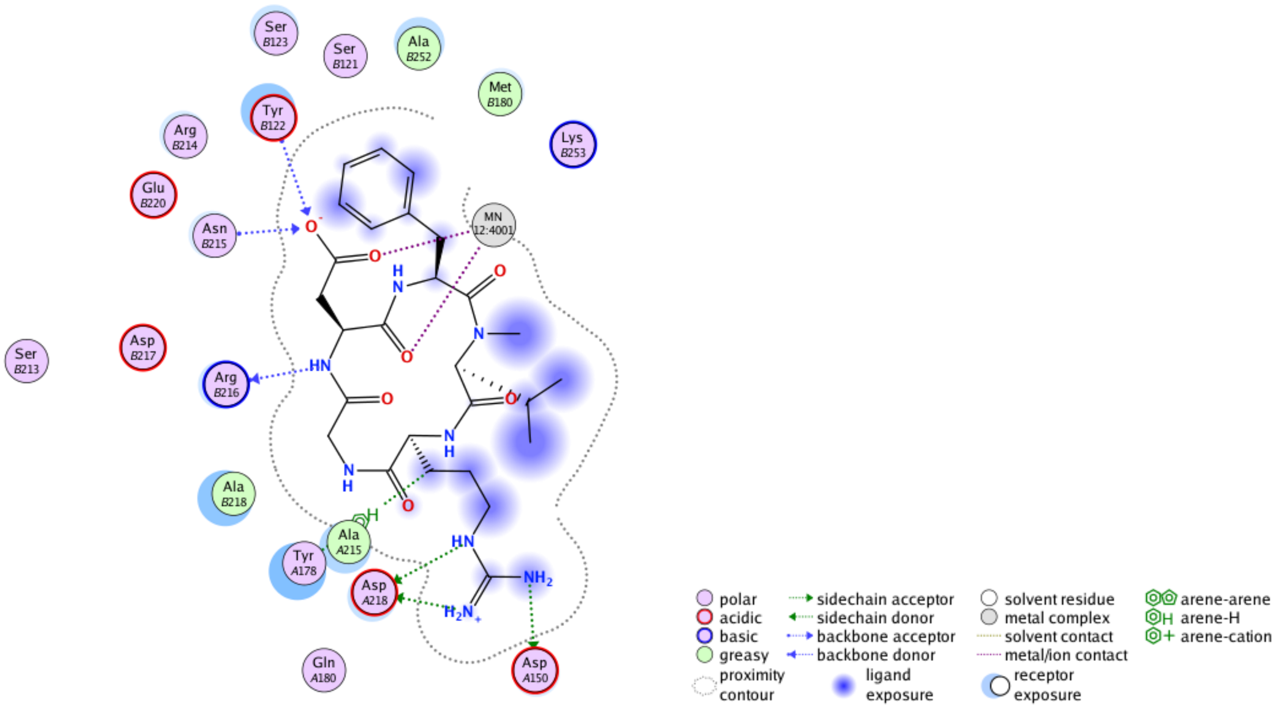

Figure S11. Crystallized iRGD ligand interactions with $\alpha_V\beta_3$ (PDB 1L5G), with color coding annotations.

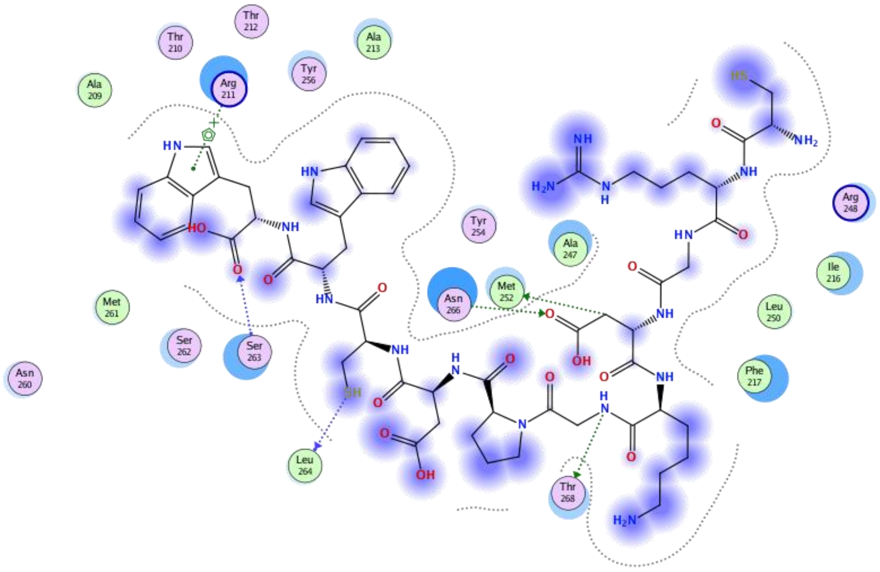

Figure S12. Predicted iRGD-WW interactions with $\alpha_V\beta_3$. The shared regions of interactions between iRGD-WW and putative interactions from iRGD are not localized at the modified C-end tryptophan doublet.

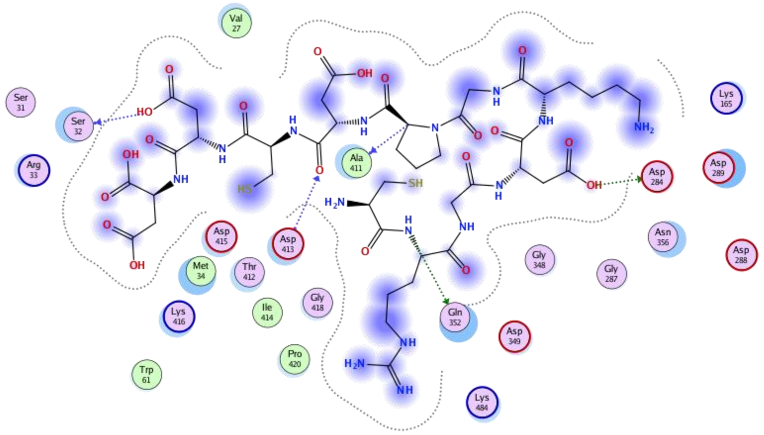

Figure S13. Predicted iRGD-DD interactions with $\alpha_V\beta_3$. The different residue positions indicate a different preferential docking site for iRGD-DD.

(a)

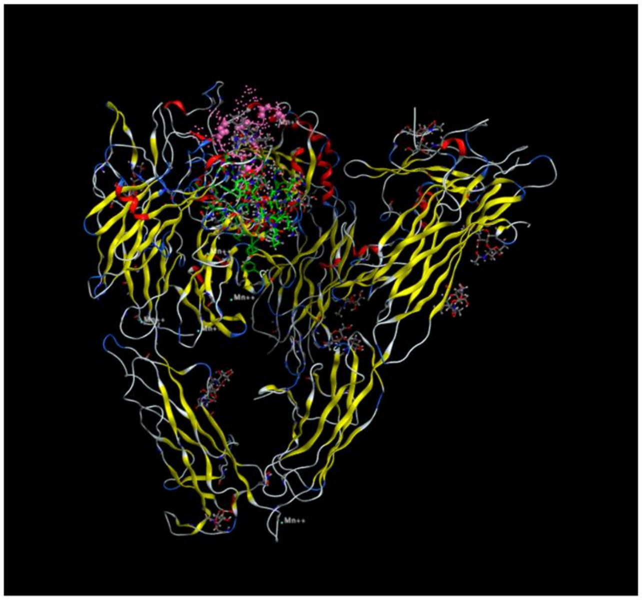

(b)

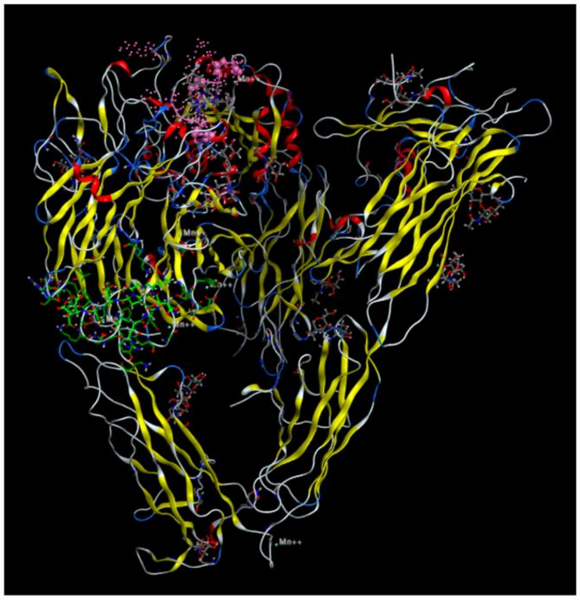

(c)

	mol	receptor	rseq	mseq	S	rmsd_refine
1		1L5G	1	1	-8.9132	3.4895
2		1L5G	1	1	-8.5606	2.1090
3		1L5G	1	1	-7.9123	3.4460
4		1L5G	1	1	-7.8877	2.9579
5		1L5G	1	1	-7.8167	2.8303
6		1L5G	1	2	-11.8721	5.3187
7		1L5G	1	2	-9.7303	3.3857
8		1L5G	1	2	-9.3591	4.0272
9		1L5G	1	2	-9.1218	2.6999
10		1L5G	1	2	-9.0106	3.9073

Figure S14. Docking predictions between designer peptides and PDB 1L5G. Panels (a) and (b) show the iRGD-WW and iRGD-DD peptides docked with $\alpha_V\beta_3$, respectively. The receptor is color coded by secondary structure, and the putative RGD binding domain is highlighted in pink, with the peptides highlighted in green. iRGD-WW shows close proximity to the putative domain, while the iRGD-DD molecule is found at the lower blades of the propeller region. (c) Raw docking S scores and poses for iRGD-DD (1-5) and iRGD-WW (6-10)
